# Supplementary material for: Current Evolutionary Dynamics of Porcine Epidemic Diarrhea Virus (PEDV) in the U.S. a Decade After Introduction
Source: Viruses. 2025 Apr 30;17(5):654. doi: 10.3390/v17050654 (PMC12115665; doi:10.3390/v17050654)
Supplement: Supplementary file 1 [file viruses-17-00654-s001.zip › viruses-3555846-supplementary.pdf]

FigureS1. Time-scaled tree of PEDV spike protein sequences from PEDV S-INDEL strains. The scale at the bottom of the tree represents the time scale in years.

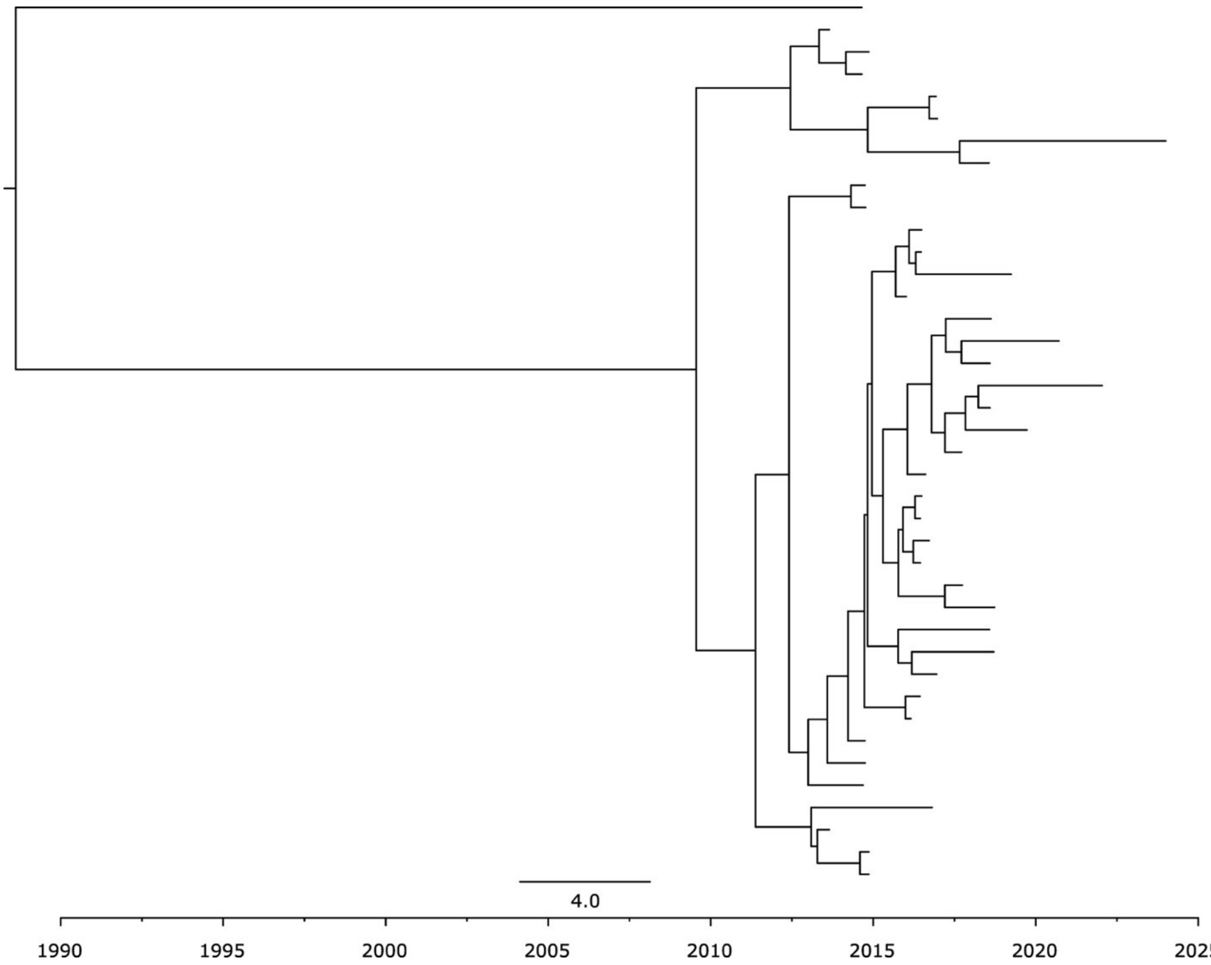

Table S1. Results of BETS (Bayesian Evaluation of Temporal Signal) Analyses The positive (log) Bayes factors obtained when comparing the model incorporating sample dates (heterochronous) to the model without sample dates (isochronous) indicate a clear temporal signal. When the correct dates were provided for the sequences, it was possible to calibrate the molecular clock. The (log) Bayes factors were calculated as the difference in the (log) marginal likelihoods of the candidate models, Model 1 (Heterochronous) vs. Model 2 (Isochronous), (log) marginal likelihoods was estimations performed using Generalized Stepping-Stone (GSS) sampling.

	BETS	
Molecular clock	Strict Clock	Relaxed clock
Non-S-INDEL-isochronous	-9,700.24	-9647.73
Non-S-INDEL-heterochronous	-9,557.55	-9499.97

log Bayes factor	142.69	147.76
Molecular clock	Strict Clock	Relaxed clock
S-INDEL-isochronous	-4502.30	-4535.76
S-INDEL-heterochronous	-4499.15	-4502.3
log Bayes factor	3.15	33.46
